# Evaluation of the RSR 3 screen ICA™ and 2 screen ICA™ as screening assays for type 1 diabetes in Sweden

**DOI:** 10.1007/s00592-022-01856-5

**Published:** 2022-02-26

**Authors:** Carina Törn, Fariba Vaziri-Sani, Anita Ramelius, Helena Elding Larsson, Sten Anders Ivarsson, Marie Amoroso, Jadwiga Furmaniak, Michael Powell, Bernard Rees Smith

**Affiliations:** 1 Department of Clinical Sciences, Lund University, Malmö, Sweden; 2 Lund, Sweden; 3 FIRS Laboratories, RSR Ltd, Llanishen, Cardiff UK; 4 Unit for Diabetes and Celiac Disease, Wallenberg Laboratory/CRC, Inga Marie Nilssons gata 53, 205 02 Malmö, Sweden

**Keywords:** GADA, IA-2A, ZnT8A, IAA, Sensitivity, Specificity

## Abstract

**Aim:**

The study aim was to evaluate the RSR 3 Screen ICA™ and 2 Screen ICA™ for detection of islet cell autoimmunity in healthy Swedish subjects and patients with newly diagnosed type 1 diabetes (T1D).

**Methods:**

3 Screen is designed for combined detection of autoantibodies to glutamic acid decarboxylase (GADA), to the islet antigen IA-2 (IA-2A) and to zinc transporter 8 (ZnT8A), while 2 Screen detects GADA and IA-2A. Serum samples from 100 T1D patients at onset and 200 healthy controls were studied.

**Results:**

3 Screen achieved 93% assay sensitivity and 97.5% specificity, while 2 Screen achieved 91% assay sensitivity and 98.5% specificity. Samples were also tested in assays for individual autoantibodies. There was only one 3 Screen positive healthy control sample (0.5%) that was positive for multiple autoantibodies (IA-2A and ZnT8A). In contrast, most of the 93 3 Screen positive patients were positive for multiple autoantibodies with 72% (67/93) positive for both GADA and IA-2A and 57% (53/93) positive for three autoantibodies (GADA, IA-2A and ZnT8A). Insulin autoantibodies (IAA, measured by radioimmunoassay) were positive in 13 patients and two healthy controls.

**Conclusion:**

3 Screen achieved high sensitivity and specificity, suitable for islet cell autoimmunity screening in a healthy population. In the case of 3 Screen positivity, further assays for GADA, IA-2A and ZnT8A are required to check for multiple autoantibody positivity, a hallmark for progression to T1D. In addition, testing for IAA in children below two years of age is warranted.

**Supplementary Information:**

The online version contains supplementary material available at 10.1007/s00592-022-01856-5.

## Introduction

Radiobinding assays (RBAs) are well established for the detection of autoantibodies to glutamic acid decarboxylase 65 (GADA) [1, 2] islet antigen-2 (IA-2A) [3, 4] and zinc transporter 8 (ZnT8A) [5, 6]. RBAs employing displacement with cold insulin are used for measuring insulin autoantibodies (IAA) [7] while islet cell autoantibodies (ICA) are determined by immunofluorescence (IF) techniques [8]. These autoantibodies are specific serological markers of type 1 diabetes (T1D) and serve as important tools for clinicians to determine the clinical classification, prediction of the need of insulin treatment, to identify subjects at risk for developing T1D and as end-points in observational studies such as The Environmental Determinants of Diabetes in the Young (TEDDY) [9], BABYDIAB [10] and TrialNet [11]. The TEDDY study has demonstrated that T1D in children up to two years of age at onset is most associated with the presence of IAA only, while GADA alone is more common after this age [9]. Well-established "in-house" RBAs for beta-cell-specific autoantibodies are being replaced by commercially available enzyme-linked immune assays (ELISA). The ELISA format provides several advantages over RBAs. These include the elimination of radioactive substances for safer handling, less environmental impact from radioactive waste disposal, and longer shelf life. The major caveat with ELISAs is that a larger volume of serum is required for the assay (25–50 µL/well as compared to 2–5 µL/well for some RBAs). The ElisaRSR™ GADAb (GADA ELISA), ElisaRSR™ IA-2 Ab Version 2 (IA-2A ELISA), and ElisaRSR™ ZnT8 Ab™ (ZnT8A ELISA) manufactured by RSR Ltd (Cardiff, UK; www.rsrltd.com) have been assessed in workshops organized by the diabetes autoantibody standardization program (DASP) and international autoantibody standardization program (IASP) [12, 13]. Furthermore, ElisaRSR™ 2 Screen ICA™ for combined measurements of GADA and IA-2A (2 Screen) and ElisaRSR™ 3 Screen ICA™ (3 Screen) for combined measurement of GADA, IA-2A and ZnT8A are also available from RSR and have been evaluated in the proficiency programs. The ELISAs for the combined measurements of autoantibodies are particularly suited for population screening purposes when the majority of samples would be expected to be negative for all two/three autoantibodies. Only the samples that score positive in the combined assays would need to be tested in the ELISAs for individual autoantibodies reducing cost, time, staff resources, and environmental impact.

The primary aim of this study was to assess the sensitivity and specificity of 2 Screen and 3 Screen ELISAs in samples representative of the Swedish population with a view to carrying out screening studies in future. In addition, the study aimed to evaluate if IAA and ICA contributed to increased sensitivity and to assess the concordance of the measurements of GADA, IA-2A and ZnT8A in the combined assays with the individual autoantibody measurements using both RBA and ELISA.

## Materials and methods

### Study design

The study included 100 patients diagnosed with T1D from May 1996 until January 2009 (median age 14.9 yrs; range 2.3–41; M/F: 59/41 = 1.44). Of these, 93 were sampled on the day of diagnosis, three were sampled within one day from diagnosis while the duration of T1D was unknown in four patients. The majority (93%) of the patients were diagnosed within the Scania region in southern Sweden while seven patients were from elsewhere in Sweden. In this study group 50 patients were children (median 8.8 years; range 2.3–14.7; M/F: 27/23 = 1.17) and 50 patients were adults (median 16.5 years; range 15.1–41; M/F: 32/18 = 1.78).

T1D was diagnosed using World Health Organization (WHO) and American Diabetes Association (ADA) criteria [14, 15].

Healthy controls (100 children and 100 adults) were from the Scania region (median age 17.3 years; range 11.9–65; M/F: 109/91 = 1.20). The median age of control children was 12.6 years; range 11.9–13.6 (M/F: 50/50 = 1.00) and the samples were obtained between January and March 1989 in conjunction with standard vaccinations. The adult samples were from healthy blood donors (median age 42.5 years; range 21–65; M/F: 59/41 = 1.44) collected in September 2008. There were no visible signs of hemolysis, lipaemic or icteric discoloration in any of the samples.

### Assays

Autoantibodies to GAD_65_, IA-2 and ZnT8 were measured using in-house RBAs with ^35^S-methionine labelled antigen. ZnT8A RBAs were carried out using ZnT8 variants at residue 325 of arginine (ZnT8-R), tryptophan (ZnT8-W), or glutamine (ZnT8-Q). In addition, RBAs with a mixture of all three ZnT8 variants (triple assay) were also carried out [16]. All samples from T1D patients and the control children were analyzed with the three separate RBA assays (ZnT8 R/W/Q). The samples from adult controls were screened with the ZnT8A triple assay and since all were negative, these samples were not tested in the individual ZnT8A variant assays.

IAA were measured by an in-house competitive RBA based on ^125^I-labelled insulin and displacement with cold insulin [17].

The intra-assay coefficient of variation (CV) for duplicates in GADA, IA-2A, ZnT8A and IAA RBAs have previously been reported [17]. In the IASP 2018 workshop the Lund University laboratory achieved sensitivity and specificity, respectively for GADA (64%, 94.4%), IA-2A (62%, 100%), ZnT8-RA (54%, 100%), ZnT8-WA (52%, 100%), ZnT8-QA (40%, 100%), IAA (18%, 96.7%), and ICA (60%, 100%).

ICA were analyzed with a two-colour immunofluorescence assay (ICA-IF) using the human pancreas as antigen [18].

The GADA, IA-2A Version 2, ZnT8A, 2 Screen and 3 Screen ELISAs in kit form from RSR were performed at Lund University according to their respective Instructions for Use (IFU, www.rsrltd.com). Results for the GADA, IA-2A and 2 Screen are expressed in NIBSC 97/550 international units (U/mL). Results for the ZnT8A and 3 Screen ELISAs are expressed in RSR arbitrary units (units/mL). Results were read on Hyperion Microreader 4 Plus (Hyperion Inc, Miami, FL, US) and Biotek Eon (Biotek Instruments, Winooski, VT. US) microplate readers. Both 450 nm and 405 nm were recorded and the 450 nm were readings used for the lower values and 405 nm to determine higher values as per IFU recommendations.

For each ELISA both a cut-off threshold derived from the receiver operator characteristic (ROC-) curve and the IFU recommended cut-off were used. The values of these two cut-offs were: 2 Screen 1.8U/mL and 4U/mL; 3 Screen 17.5units/mL and 20units/mL; GADA 5.9U/mL and 5.0U/mL; IA-2A 9.7U/mL and 7.5U/mL; ZnT8A 12.9 units/mL and 15units/mL, (ROC-curve and IFU, respectively in all cases). Results for 3 Screen were also expressed as an index value based on sample OD as a percentage of a kit reference preparation OD with the IFU recommended cut-off index of 30.

RSR ELISAs' reported sensitivity and specificity in IASP 2020 were: for 2 Screen (96%, 98.9%), 3 Screen (96%, 100%), GADA (88%, 98.9%), IA-2A (72%, 100%), and ZnT8A (74%, 98.9%), respectively.

### Data analysis

For each assay, sensitivity and specificity were calculated using previously defined thresholds for the RBAs and thresholds from ROC-curves and IFUs for the ELISAs. The area under the ROC-curve (AUC) with 95% confidence interval (CI) was calculated assuming a non-parametric distribution of autoantibody results. An AUC of 1.0 would indicate that the assay achieved 100% accuracy in identifying disease, while an AUC of 0 would indicate that the assay gave positive results for control subjects and negative results for patients.

Kappa was used to estimate the agreement between different assays for the measurement of autoantibodies [19]. A Kappa value of 1.0 indicates perfect agreement, whereas a Kappa value of less than 0 indicates agreement equivalent to chance. The Spearman rank correlation test was used between variables with a non-normal distribution of values (*r*_*s*_). Mann–Whitney *U*-test was used to compare antibody levels in patient and control samples. Chi-square test was used to compare frequencies of autoantibody positive and negative results for each assay. Two-tailed p values less than 0.05 were considered significant.

## Results

### Sensitivity and specificity

The results from RBAs, ICA-IF and ELISAs are summarized in Tables 1 and 2. Using the ROC-threshold, 2 Screen achieved 94% sensitivity at 97% specificity, while lower sensitivity of 91% at higher specificity of 98.5% using the IFU-threshold. 3 Screen achieved slightly lower sensitivity (93%) at 97% specificity, when the cut-off from ROC-curve was applied. Using the IFU-cut off, 3 Screen achieved 93% sensitivity at 97.5% specificity with both units/mL or index cut-offs). Two patient samples were positive in 3 Screen but not in 2 Screen with the IFU cut-offs. One of these was positive only in the ZnT8A ELISA and the other in the ZnT8A and IA-2A ELISAs. Five of the 93 3 Screen positive T1D samples (5.4%) were positive for only one autoantibody by ELISA, 35 (38%) were positive for two different autoantibodies and 53 (57%) were positive for all three autoantibodies by ELISAs. Among the seven 3 Screen negative T1D samples, one was positive by GADA ELISA; one by IA-2A ELISA and another by ZnT8A ELISA (all at low levels).

**Table 1:** Combinations of beta cell specific autoantibodies in 100 patients with type 1 diabetes. Samples were analyzed with six radiobinding assays (RBAs) and with five ELISAs assays

Combinations of autoantibodies	N (%)	M/F	N (%)	M/F
*Sensitivity when analyzed with RBAs and ICA*				
Any autoantibody (GADA or IA-2A or ZnT(R)A or ZnT8(W)A or ZnT8(Q)A)	91 (91%)	54/37 (1.46)		
Any autoantibody (GADA or IA-2A or ZnT(R)A or ZnT8(W)A or ZnT8(Q)A or IAA or ICA)	91 (91%)	54/37 (1.46)		
IA-2A	78 (78%)	45/33 (1.36)		
GADA	77 (77%)	44/33 (1.33)		
ZnT8RA	51 (51%)	28/23 (1.22)		
ZnT8WA	43 (43%)	26/17 (1.53)		
ZnT8QA	33 (33%)	19/14 (1.36)		
Any ZnT8A (ZnT8(R)A/ZnT8(W)A/ZnT8(Q)A)	61 (61%)	35/26 (1.35)		
IAA	13 (13%)	7/6 (1.16)		
ICA-IF	79 (79%)	45/34 (1.32)		
GADA and IA-2A and ZnT(R)A and ZnT8(W)A and ZnT8(Q)A	20 (20%)	11/9 (1.22)		
GADA in those not positive for IA-2A	12 (12%)	9/3 (3.00)		
ZnT8(R)A/ZnT8(W)A/ZnT8(Q)A in those not positive for GADA or IA-2A	1 (1%)	0/1 (NA)		
Negative for all RBA and IF (GADA, IA-2A, ZnT8A, IAA, ICA)	9 (9%)	5/4 (1.25)		

Sensitivity when analyzed with ELISAs	Threshold ROC-curve		Threshold IFU	
Any ELISA (3 Screen by units/mL or Index or 2 Screen or GADA or IA-2A or ZnT8A)	96 (96%)	56/40 (1.40)	96 (96%)	56/40 (1.40)
2 Screen	94 (94%)	55/39 (1.41)	91 (91%)	53/38 (1.39)
3 Screen (units/mL)	93 (93%)	54/39 (1.38)	93 (93%)	54/39 (1.38)
3 Screen (Index)	N/A	N/A	93 (93%)	54/39 (1.38)
GADA	83 (83%)	45/38 (1.18)	84 (84%)	46/38 (1.21)
IA-2A	74 (74%)	43/31 (1.39)	78 (78%)	45/33 (1.36)
ZnT8A	75 (75%)	44/31 (1.42)	75 (75%)	44/31 (1.42)
3 Screen and 2 Screen and GADA and IA-2A and ZnT8A (5 ELISAs)	52 (52%)	30/22 (1.36)	53 (53%)	30/23 (1.30)
2 Screen and GADA	83 (83%)	45/38 (1.18)	82 (82%)	45/37 (1.22)
IA-2A in those not positive for GADA	10 (10%)	8/2 (4.00)	11 (11%)	9/2 (4.50)
2 Screen and GADA or IA-2A	92 (92%)	53/39 (1.36)	91 (91%)	53/38 (1.39)
ZnT8A in those not GADA positive or IA-2A positive	3 (3%)	3/0 (NA)	1 (1%)	1/0 (NA)
Positive GADA alone	0	0	1 (1%)	1/0 (NA)
Positive IA-2A alone	1 (1%)	0/1 (NA)	1 (1%)	0/1 (NA)
Positive ZnT8A alone	1 (1%)	1/0 (NA)	1 (1%)	1/0 (NA)
Positive 2 Screen, 3 Screen and only ZnT8A	2 (2%)	2/0 (NA)	0 (0%)	0 (0)
Negative in all ELISAs	4 (4%)	3/1 (3.00)	4 (4%)	3/1 (3.00)

ICA was analysed with immunofluorescence (IF). For ELISAs both the threshold for positivity derived from the Receiver Operating Characteristic Curve (ROC-curve) and the Instructions for Use (IFU) were used

**Table 2:** Combinations of beta cell specific autoantibodies in 200 healthy controls. Samples were analyzed with six radiobinding assays (RBAs) and five ELISAs

Combinations of autoantibodies	N (%)	M/F	N (%)	M/F
*Combinations when analysed with RBA and ICA*				
Any autoantibody (GADA or IA-2A or ZnT(R)A or ZnT8(W)A or ZnT8(Q)A)	10 (5%)	4/6 (0.67)		
Any autoantibody (GADA or IA-2A or ZnT(R)A or ZnT8(W)A or ZnT8(Q)A or IAA or ICA)	12 (6%)	4/8 (0.50)		
IA-2A	0 (0%)	0		
GADA	8 (4%)	3/5 (0.60)		
ZnT8(R)A	1 (0.5%)	0/1 (NA)		
ZnT8(W)A	0 (0%)	0		
ZnT8(Q)A	2 (1%)	1/1 (1.00)		
Any ZnT8A (ZnT8(R)A/ZnT8(W)A/ZnT8(Q)A)	2 (1%)	1/1 (1.00)		
IAA	2 (1%)	0/2 (NA)		
ICA-IF	0 (0%)	0		
ZnT8(R)A or ZnT8(W)A or ZnT8(Q)A in those not positive for GADA or IA-2A	2 (1%)	1/1 (1.00)		
Negative for all RBA and IF (GADA, IA-2A, ZnT8A, IAA, ICA)	188 (94%)	105/183 (0.57)		

Combinations when analyzed with ELISA assays	Threshold ROC-curve		Threshold IFU	
Any ELISA (3 Screen (units/mL) or 2 Screen or GADA or IA-2A or ZnT8A)	16/200 (8%)	11/5 (2.20)	14 (7%)	10/4 (2.50)
Any ELISA (3 Screen (Index) or 2 Screen or GADA or IA-2A or ZnT8A)	14/200 (7%)	10/4 (2.50)	13 (6.5%)	9/4 (2.225)
2 Screen	6/200 (3%)	2/4 (0.500)	3 (1.5%)	2/1 (2.00)
3 Screen (units/mL)	6/200 (3%)	3/3 (1.00)	5 (2.5%)	3/2 (1.50)
3 Screen (Index)	NA	NA	5 (2.5%)	2/3 (0.67)
GADA	4/200 (2%)	2/2 (1.00)	4/200 (2%)	2/2 (1.00)
IA-2A	5/200 (2.5%)	4/1 (4.00)	6/200 (3%)	4/2 (2.00)
ZnT8A	5/200 (2.5%)	4/1 (4.00)	4/200 (2%)	3/1 (3.00)
3 Screen and 2 Screen and GADA and IA-2A and ZnT8A (5 ELISAs)	0	NA	0	NA
2 Screen and GADA	4/200 (2%)	2/2 (1.00)	3/200 (1.5%)	2/1 (2.00)
IA-2A in those not positive for GADA	5/200 (2.5%)	4/1 (4.00)	6/200 (3%)	4/2 (2.00)
2 Screen and GADA or IA-2A	5/200 (2.5%)	2/3 (0.67)	3/200 (1%)	2/1 (2.00)
ZnT8A in those not GADA positive or IA-2A positive	4/200 (2%)	4/0 (NA)	3/200 (3%)	3/0 (NA)
Positive GADA alone	0/200 (0%)	0/0 (NA)	1/200 (0.5%)	0/1 (NA)
Positive IA-2A alone (3 Screen Index cut off)	4/200 (2%)	4/0 (NA)	5/200 (2.5%)	4/1 (4.00)
Positive IA-2A alone (3 Screen units/mL cut off)	3/200 (1.5%)	3/0 (NA)	4/200 (2%)	3/1 (3.00)
Positive ZnT8 alone	4/200 (2%)	4/0 (NA)	3/200 (1.5%)	3/0 (NA)
Positive 2 Screen, 3 Screen (units/mL or Index), IA-2A and ZnT8A (4 ELISAs)	1/200 (0.5%)	0/1 (NA)	0	NA
Positive 2 Screen, 3 Screen (units/mL or Index), and GADA (3 ELISAs)	2/200 (1%)	1/1 (1.00)	3/200 (1.5%)	2/1 (2.00)
Positive 2 Screen and GADA (negative 3 Screen units/mL) (2 ELISAs)	2/200 (0.5%)	1/1 (1.00)	1/200 (0.5%)	1/0 (NA)
Positive 2 Screen and GADA (negative 3 Screen Index) (2 ELISAs)	NA	NA	0	NA
Positive 3 Screen (units/mL or Index) and IA-2A (negative 2 Screen) (2 ELISAs)	1/200 (0.5%)	1/0 (NA)	1/200 (1%)	1/0 (NA)
Negative in all ELISA (3 Screen units/mL)	184/200 (92%)	98/86 (1.14)	186/200 (93.0%)	99/87 (1.14)
Negative in all ELISA (3 Screen Index)	NA	NA	187/200 (93.5%)	100/87 (1.15)

ICA was analyzed with immunofluorescence (IF). For ELISAs both the threshold for positivity derived from the Receiver Operating Characteristic Curve (ROC-curve) and the Instructions for Use (IFU) were used

Using the ELISAs for any of the three specific autoantibodies, most of the patients (83% using the ROC-threshold and 84% using the IFU-threshold) were positive in the GADA ELISA. The addition of results from the IA-2A ELISA increased the sensitivity by 8% to 91% using the ROC-threshold and by 11% to 95% using the IFU cut-off. The addition of results from the ZnT8A ELISA increased the sensitivity by another 3% to 94% using the ROC-threshold and by 1% to 96% using the IFU-threshold which is equivalent to the sensitivity of 2 Screen (94%; ROC-threshold) and slightly higher than the sensitivity of 3 Screen (93%) using either threshold. All seven patients with T1D that were negative by the 3 Screen assay (IFU-cut off) were also negative by 2 Screen.

All ELISAs achieved excellent AUC. The highest AUC was achieved by 2 Screen while the lowest was achieved by ZnT8A [Supplementary Fig. 1].

For RBAs, IA-2A achieved the highest sensitivity of 78%. The addition of GADA RBA results increased the sensitivity by 12% to 90%, while the addition of ZnT8A RBA (any of RWQ), only increased sensitivity by 1% to 91%. In contrast, the addition of IAA and ICA-IF results did not confer an increase of sensitivity above that achieved using the other assays (Table 1).

Only four patients were negative for all assays (using either cut-offs in the case of the ELISAs) while a total of 174 healthy controls (87%) were negative in all assays using the ROC-cut-off and 177 (88%) with the IFU-cut-off (Table 3). 95% of patients were positive by more than one assay using the ROC-threshold and 94% using the IFU cut-off, whereas only seven healthy controls (3.5%) were positive by more than one assay (four by two assays and three by four assays) using the ROC-threshold while 19 controls were positive by only one assay (Table 3). Six healthy controls (3.0%) were positive by more than one assay, using the IFU-threshold, while 9% were positive by only one assay (Table 3). The ELISA results for the 3 Screen positive control samples (either units/mL or index cut-off) are detailed in Supplementary Table 1. The small number of apparently discrepant results were all close to the cut-offs in the respective assays.

**Table 3:** Positivity for autoantibodies in patients with type 1 diabetes (*n* = 100) and in healthy controls (*n* = 200) tested in 12 different assays using both the threshold for positivity derived from the Receiver Operating Characteristic Curve (ROC-curve) and the Instructions for Use (IFU where applicable), the units/mL cut off was used in the case of 3 Screen

Number of assays in which sample was positive	Patients with type 1 diabetes	Healthy controls	Patients with type 1 diabetes	Healthy controls
	Threshold ROC-curve		Threshold IFU	
	Number of samples (%)	Number of samples (%)	Number of samples (%)	Number of samples (%)
0	4 (4)	174 (87)	4 (4)	177 (88)
1	1 (1)	19 (9.5)	2 (2)	18 (9)
2	2 (2)	4 (2)	1 (1)	2 (1.0)
3	2 (2)	0 (0)	1 (1)	1 (0.5)
4	2 (2)	3 (1.5)	3 (3)	2 (1)
5	7 (7)	0 (0)	6 (6)	0 (0)
6	4 (7)	0 (0)	5 (5)	0 (0)
7	13 (13)	0 (0)	13 (13)	0 (0)
8	16 (16)	0 (0)	16 (16)	0 (0)
9	18 (18)	0 (0)	18 (18)	0 (0)
10	11 (11)	0 (0)	10 (10)	0 (0)
11	16 (16)	0 (0)	17 (17)	0 (0)
12	4 (4)	0 (0)	4 (4)	0 (0)

None of the 18 controls positive in only one assay was positive by 2 Screen, only one by 3 Screen, six were positive only in the GADA RBA, five were positive only in the IA-2A ELISA, three were positive only in the ZnT8A ELISA, and one subject was positive only in the GADA ELISA. Four healthy subjects were positive only by RBAs but not in the respective ELISAs, two subjects were positive only for ZnT8QA and two were positive only in the IAA assay. Positivity for multiple autoantibodies was rare among the controls with only one subject positive in both IA-2A and ZnT8A ELISAs.

All ELISAs achieved good intra-assay precision with CVs for duplicates for positive samples: for 2 Screen 3.2%, 3 Screen 5.5%, GADA ELISA 5.5%, IA-2A ELISA 8.1% and ZnT8A ELISA 8.1%.

### Agreement of pairs of autoantibodies measured with RBA and ELISA assays

There was good agreement for six pairs of autoantibody assays using the IFU-thresholds for ELISAs. The closest agreement between any two assays was found for 2 Screen and 3 Screen (Kappa = 0.97; Standard Error = 0.015). The best agreement between pairs of autoantibodies measured using different techniques was found for IA-2A RBA and IA-2A ELISA (Kappa = 0.96; Standard Error = 0.019) (Supplementary Table 2).

The correlation between different assays ranged from r_*s* =_ 0.949 for GADA RBA vs GADA ELISA (*n* = 76; *p* ˂ 0.001) to *r*_*s*_ = 0.244 (*n* = 74; *p* = 0.003) for 3 Screen vs ZnT8A ELISA (*p* = 0.001) (Supplementary Figs. 2 and 3).

### Levels of autoantibodies in autoantibody positive samples

There were no differences in autoantibody levels between males and females for any assay irrespective of which cut-off was chosen. There were differences between patients below 15 and 15 years old and above for 2 Screen and 3 Screen. In 2 Screen, levels were higher in patients aged 15 years and above as compared with younger patients (*p* = 0.009) using the ROC-threshold. A similar result was obtained if the IFU-cut-off was used, (*p* = 0.028). For 3 Screen, autoantibody levels were also higher in the older age group (*p* = 0.022) using either the ROC-threshold or the IFU-threshold (Supplementary Table 3).

## Discussion

In this study comprising six RBAs, one ICA IF and five ELISAs to detect beta-cell specific autoantibodies in 100 patients and 200 healthy controls we found that 3 Screen was the most versatile assay for screening purposes showing the highest sensitivity (93%) and high specificity (97.5%). The combined detection of three autoantibodies in 3 Screen resulted in slightly increased sensitivity compared to the 2 Screen assay (93% and 91%, respectively) although 2 Screen had slightly higher specificity. Assay sensitivity for 2 Screen and for 3 Screen was superior to those of any RBA.

The ELISAs ROC and IFU defined cut-offs produced similar results suggesting the IFU cut-offs were suitable for the samples tested. 3 Screen used without calibrators with results expressed as index has an advantage as more samples can be tested per kit reducing the time and cost of population screening. Using units/mL or index cut-offs had little effect on 3 Screen results.

T1D patients are usually positive for more than one autoantibody [20] and in our study, 95% of patients were positive in two to 12 assays using ROC-curve cut-offs compared with 3.5% of healthy controls.

The GADA ELISA identified more patients diagnosed with T1D as positive (84%; IFU-cut off) versus GADA RBA (77%) with the majority (76%) of patients positive in both GADA assays, while eight patients were positive for GADA by ELISA only and one by GADA RBA only. The discrepancy between GADA ELISA and RBA could be due to differences in conformation of antigens produced in different expression systems, differences in antibody binding to liquid phase antigen (RBA) compared to solid-phase antigen (ELISA) and/or differences in antibody affinity [21, 22]. Furthermore, it has been reported that GADA found in healthy subjects recognize a broader repertoire of epitopes on the GAD molecule compared to T1D associated autoantibodies [23].

The ZnT8A ELISA (ZnT8R and ZnT8W variants) identified more (75%) T1D patients as positive compared to the triple assay (ZnT8R, ZnT8W and ZnT8Q) RBA (61%). This discrepancy could be due to the differences in antigens from different expression systems and/or the assay formats. The IA-2A ELISA and RBA identified an equal number of patients as positive (78%; IFU cut-off for ELISA).

3 Screen is particularly suitable for population screening. Although serum samples are preferred for analysis of autoantibodies, also Ca2+-treated EDTA plasma can be used with reliable results [24, 25]. Capillary samples can also be used for the measurement of autoantibodies using 3 Screen with robust results [26]. Addition of haemoglobin, biotin, intralipid, or bilirubin to samples did not markedly affect results suggesting the assay is not prone to interference from such substances [27www.rsrltd.com].

Population screening strategies with 3 Screen could use a cut-off on the basis of percentiles (rather than the cut-off recommended in the IFU), to determine the proportion of samples which should be tested subsequently for individual autoantibodies [26]. This would have the advantage of setting the cut-off for the actual test population.

3 Screen can be compared with other assays which detect multiple autoantibodies. In particular, a multiplex Electro Chemi Luminescence based assay (ECLIA) [28], which has similar sensitivity and specificity to 3 Screen. Also, a multiplex Luciferase Immunoprecipitation Assay (LIPS) showed similar sensitivity and specificity to 3 Screen in IASP 2020 (IASP 2020 Cumulative Performance Summary).

The strength of our study is that most patients (93/100) were sampled from the same area and comprised both pediatric and adult cases with T1D. To match the demographics of T1D subjects, the control population consisted of both healthy children and adults with serum samples from both stored under the same conditions (− 20 °C) for similar times. A limitation of the study is that only 100 T1D patients and 200 healthy controls were included and larger study groups would have strengthened the application of the results for assessment of the Swedish population in general. Another limitation is that for practical and ethical reasons there were no healthy controls below 12 years of age. A further limitation for population screening is that IAA are not included in 3 Screen ELISA. IAA positive only samples could be 3 Screen negative and a 3 Screen only strategy would not be preferred for children below two years of age.

In conclusion, this study showed excellent performance for 3 Screen with high assay sensitivity and specificity suitable for screening purposes in healthy populations to detect three key beta-cell specific autoantibodies. Further analyses using individual antibody ELISAs plus IAA RBA are required to identify multiple autoantibody positives with high risk of progression to T1D. Samples negative by 3 Screen assays would not normally require further testing. A strategy of testing by 3 Screen plus IAA would be more suitable for very young children.

## Supplementary Information

Below is the link to the electronic supplementary material.

Supplementary file1 (DOCX 45 kb)

Supplementary file2 (PPTX 355 kb)

## Data Availability

Anonymized Data is available on request.

## Code availability

IBM SPSS Statistics 27 (Armonk, New York, US) was used for the statistical calculations.
