# Supplementary material for: Evaluation of the RSR 3 screen ICA™ and 2 screen ICA™ as screening assays for type 1 diabetes in Sweden
Source: Acta Diabetol. 2022 Feb 26;59(6):773–81. doi: 10.1007/s00592-022-01856-5 (PMC9085662; doi:10.1007/s00592-022-01856-5)
Supplement: Supplementary file 1 — Supplementary file1 (DOCX 45 kb) [file 592_2022_1856_MOESM1_ESM.docx]

**Supplementary Table 1** ELISA results for healthy control samples positive by 3 Screen (units/mL or Index IFU cut-off)

|  | 3 Screen | | 2 Screen | GADA ELISA | IA-2A ELISA | ZnT8A ELISA |
| --- | --- | --- | --- | --- | --- | --- |
| Positive controls | units/mL  (cut off ≥20 .0) | Index  (cut off ≥30) | U/mL  (cut off ≥4.0) | U/mL  (cut off ≥5.0) | U/mL  (cut off ≥7.5) | units/mL  (cut off ≥15.0) |
| 1 | 22.9 | 18.3 | 1.6 | 1.3 | 11.3 | 1.6 |
| 2 | 14.7 | 35.3 | 4.3 | 6.0 | 1.3 | 1.4 |
| 3 | 23.1 | 15.6 | 1.3 | 2.3 | 1.4 | 0.3 |
| 4 | 15.9 | 41.1 | 3.1 | 10.5 | 2.2 | 2.2 |
| 5 | 41.0 | 56.8 | 2.0 | 0.3 | 18.7 | 103.0 |
| 6 | 1163.1 | 496.6 | 184.6 | 454.9 | 0.8 | 0.6 |
| 7 | 943.2 | 558.8 | 167.3 | 337.2 | 0.7 | 0.2 |

5 out of 200 healthy controls were positive using 3 Screen units/mL cut-off and 5 out of 200 healthy controls were positive using Index IFU cut-off.

**Supplementary Table 2** Agreement of pairs of autoantibodies measured with radiobinding assays (RBAs) and ELISAs using both the threshold for positivity derived from the Receiver Operating Characteristic Curve (ROC-curve) and the Instructions for Use (IFU) in 100 newly diagnosed patients with type 1 diabetes and 200 healthy controls. The units/mL cut-off was used in the case of 3 Screen.

|  | Threshold ROC-curve | | Threshold IFU | |
| --- | --- | --- | --- | --- |
| Assay 1 vs Assay2 | Rank* | Kappa (SE) | Rank | Kappa (SE) |
| RBAs vs ELISAs |  |  |  |  |
| IA-2A RBA vs IA-2A ELISA | 1 | 0.89 (0.030) | 1 | 0.96 (0.019) |
| GADA RBA vs GADA ELISA | 2 | 0.87 (0.032) | 2 | 0.86 (0.032) |
| ZnT8(R)A RBA vs ZnT8A ELISA | 3 | 0.69 (0.049) | 3 | 0.70 (0.049) |
| ZnT8(W)A RBA vs ZnT8A ELISA | 4 | 0.59 (0.055) | 4 | 0.60 (0.055) |
|  |  |  |  |  |
| ELISAs |  |  |  |  |
| 2 Screen vs 3 Screen | 1 | 0.95 (0.020) | 1 | 0.97 (0.015) |
| 2 Screen vs GADA | 2 | 0.90 (0.027) | 2 | 0.90 (0.027) |
| 3 Screen vs GADA | 3 | 0.86 (0.032) | 3 | 0.89 (0.029) |
| 3 Screen vs IA-2A | 4 | 0.78 (0.039) | 4 | 0.81 (0.037) |
| 3 Screen vs ZnT8A | 5 | 0.77 (0.040) | 5 | 0.80 (0.038) |
| 2 Screen vs IA-2A | 6 | 0.76 (0.041) | 6 | 0.79 (0.039) |

Note: *Ranked by agreement to identify the respective autoantibody (Kappa).

**Supplementary Table 3** Levels of autoantibodies in autoantibody positive patients with type 1 diabetes and frequency of autoantibody positive patients. Levels of autoantibodies and frequencies were tested for significance using Mann-Whitney U-test (levels) and Chi-square test (frequencies). For ELISAs both the threshold for positivity derived from the Receiver Operating Characteristic Curve (ROC-curve) and the Instructions for Use (IFU) were used.

| Median levels; range; U/mL | | | | | | | | | | | | | | |
| --- | --- | --- | --- | --- | --- | --- | --- | --- | --- | --- | --- | --- | --- | --- |
| Assay | All patients | | Men | | Women | | P  (M-W)  (M-W) | P χ^2^ |  | ˂15 yrs |  | ≥15yrs | P  (M-W)  (Age) | P χ^2^ |
|  | (n) |  | (n) |  | (n) |  |  |  | (n) |  | (n) |  |  |  |
| RBA assays |  |  |  |  |  |  |  |  |  |  |  |  |  |  |
| GADA | (77) | 506; 40.4-2670 | (44) | 457; 43.7-2670 | (33) | 516; 40.4-2346 | 0.943 | 0.490 | (37) | 376; 40-2670 | (40) | 532; 44-2511 | 0.370 | 0.635 |
| IA-2A | (78) | 368; 6.80-469 | (45) | 411; 6.80-468 | (33) | 303; 7.47-469 | 0.123 | 0.617 | (41) | 376; 7.47-459 | (37) | 360; 6.80-469 | 0.499 | 0.470 |
| ZnT8(R)A | (51) | 722; 74.0-1100 | (28) | 708; 88-1100 | (23) | 722; 74.0-1100 | 0.790 | 0.395 | (27) | 744; 74-1100 | (24) | 410; 88-1100 | 0.111 | 0.548 |
| ZnT8(W)A | (43) | 745; 79-1100 | (26) | 736; 79.0-1100 | (17) | 770; 203-1100 | 0.594 | 0.796 | (21) | 770; 106-1100 | (22) | 736; 79-1100 | 0.637 | 0.840 |
| ZnT8(Q)A | (33) | 419; 104-1021 | (19) | 475; 131-943 | (14) | 402; 104-1021 | 0.679 | 0.839 | (18) | 422; 122-915 | (15) | 356; 104-1021 | 0.957 | 0.523 |
| IAA | (13) | 17.6; 6.74-194 | (7) | 42.; 6.83-194 | (6) | 14.1; 6.74-48.3 | 0.295 | 0.685 | (6) | 10.9; 6.74-76.1 | (7) | 28; 7.41-194 | 0.366 | 1.00 |
|  |  |  |  |  |  |  |  |  |  |  |  |  |  |  |
| IF assay |  |  |  |  |  |  |  |  |  |  |  |  |  |  |
| ICA | (79) | 74.0; 6.00-2719 | (45) | 74.0; 6.00-2719 | (34) | 34.0; 8.00-705 | 0.369 | 0.422 | (44) | 71.0; 8.00-974 | (35) | 116; 6.00-2719 | 0.076 | 0.048 |
|  |  |  |  |  |  |  |  |  |  |  |  |  |  |  |
| ELISA assays; Threshold from ROC-curve | | | | | | | | | | | | | | |
| 2 Screen | (94) | 188; 2.20-394 | (55) | 187; 2.20-394 | (39) | 188; 3.40-383 | 0.640 | 1.00 | (48) | 183; 2.20-248 | (46) | 220; 7.39-394 | 0.009 | 0.678 |
| 3 Screen | (93) | 1598; 32.0-3544 | (54) | 1635; 32.0-3544 | (39) | 1580; 39.2-2811 | 0.794 | 0.697 | (47) | 1571; 35.3-1872 | (46) | 1787; 32.0-3544 | 0.022 | 1.000 |
| GADA | (83) | 342; 6.47-2100 | (45) | 405; 6.59-2100 | (38) | 196; 6.47-2100 | 0.597 | 0.034 | (41) | 222; 6.47-2100 | (42) | 683; 8.17-2100 | 0.178 | 1.000 |
| IA-2A | (74) | 1067; 10.2-1935 | (43) | 1154; 12.8-1935 | (31) | 764; 10.2-1908 | 0.673 | 0.760 | (38) | 1156; 50.6-1906 | (36) | 984; 10.2-1935 | 0.393 | 0.648 |
| ZnT8A | (75) | 890; 17.0-1711 | (44) | 879; 17.9-1711 | (31) | 976; 17.0-1696 | 0.966 | 0.907 | (37) | 976; 17.0-1508 | (38) | 844; 17.9-1711 | 0.351 | 0.817 |
|  |  |  |  |  |  |  |  |  |  |  |  |  |  |  |
| ELISA assays; Threshold from IFU | | | | | | | | | | | | | | |
| 2 Screen | (91) | 189; 5.40-394 | (53) | 189; 9.30-394 | (38) | 190; 5.40-383 | 0.554 | 0.733 | (45) | 187; 5.40-248 | (46) | 220; 7.39-394 | 0.028 | 1.000 |
| 3 Screen (units/mL) | (93) | 1598; 32.0-3544 | (54) | 1635; 32.0-3544 | (39) | 1580; 39.2-2811 | 0.794 | 0.697 | (47) | 1571; 35.3-1872 | (46) | 1787; 32.0-3544 | 0.022 | 1.000 |
| 3 Screen (Index) | (93) | 519; 44.0-704 | (54) | 514; 44.0-704 | (39) | 522; 63.1-697 | 0.703 | 0.697 | (47) | 538; 63.1-704 | (46) | 493; 44.0-655 | <0.001 | 1.000 |
| GADA | (84) | 291; 5.77-2100 | (46) | 374: 5.77-2100 | (38) | 196; 6.47-2100 | 0.728 | 0.056 | (41) | 222; 6.47-2100 | (43) | 621; 5.77-2100 | 0.255 | 0.786 |
| IA-2A | (78) | 969; 7.92-1935 | (45) | 1026; 8.22-1935 | (33) | 733; 7.92-1908 | 0.610 | 0.617 | (39) | 1124; 8.22-1906 | (39) | 896; 7.92- 1935 | 0.240 | 1.000 |
| ZnT8A | (75) | 890; 17.0-1711 | (44) | 879; 17.9-1711 | (31) | 976; 17.0-1696 | 0.966 | 0.907 | (37) | 976; 17.0-1508 | (38) | 844; 17.9-1711 | 0.351 | 1.000 |
