# Supplementary material for: Evaluation of the RSR 3 screen ICA™ and 2 screen ICA™ as screening assays for type 1 diabetes in Sweden
Source: Acta Diabetol. 2022 Feb 26;59(6):773–81. doi: 10.1007/s00592-022-01856-5 (PMC9085662; doi:10.1007/s00592-022-01856-5)
Supplement: Supplementary file 2 — Supplementary file2 (PPTX 355 kb) [file 592_2022_1856_MOESM2_ESM.pptx]

## Slide 1
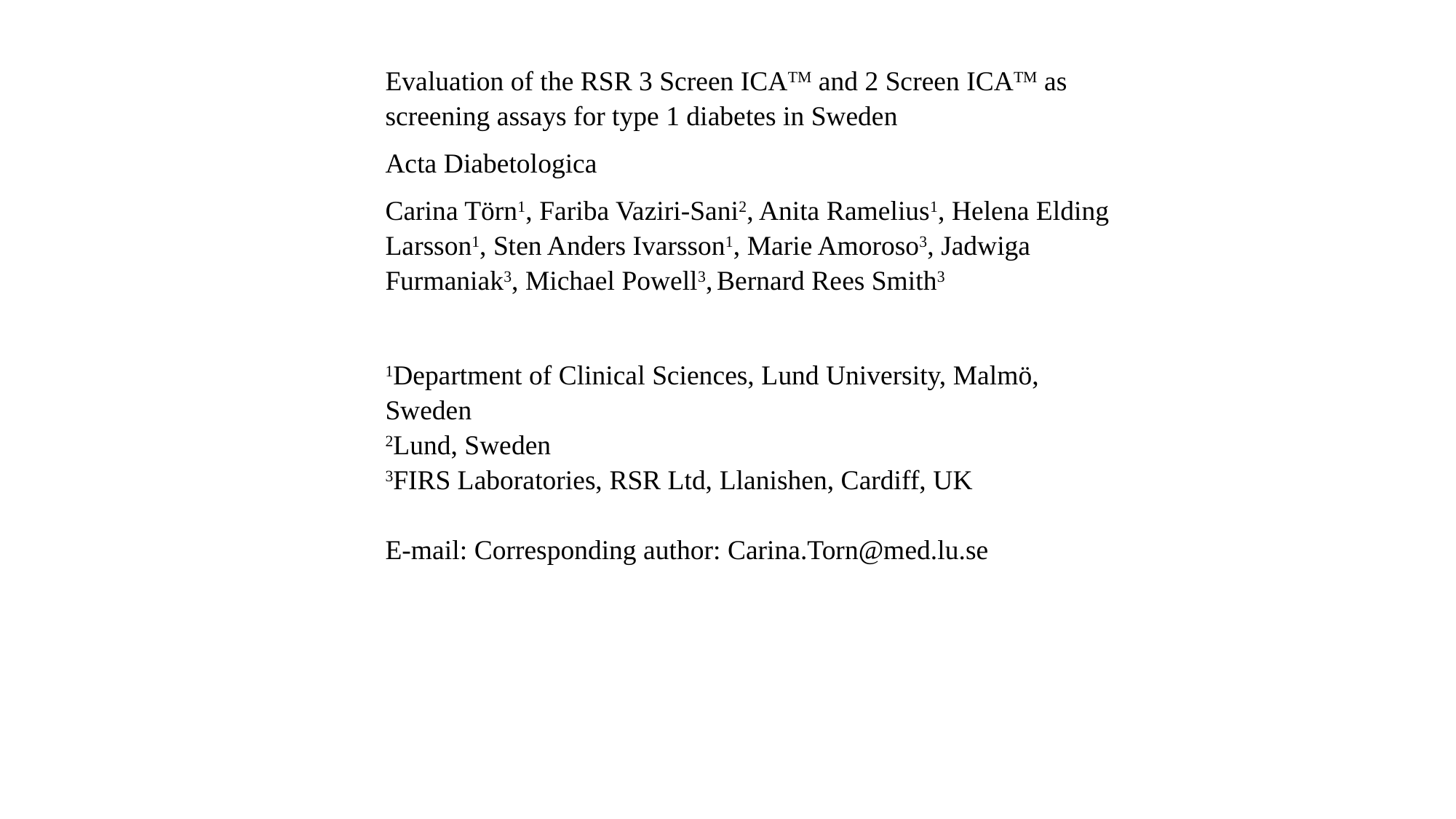

Evaluation of the RSR 3 Screen ICATM and 2 Screen ICATM as screening assays for type 1 diabetes in Sweden
Acta Diabetologica
Carina Törn1, Fariba Vaziri-Sani2, Anita Ramelius1, Helena Elding Larsson1, Sten Anders Ivarsson1, Marie Amoroso3, Jadwiga Furmaniak3, Michael Powell3, Bernard Rees Smith3
1Department of Clinical Sciences, Lund University, Malmö, Sweden
2Lund, Sweden
3FIRS Laboratories, RSR Ltd, Llanishen, Cardiff, UK
E-mail: Corresponding author: Carina.Torn@med.lu.se

## Slide 2
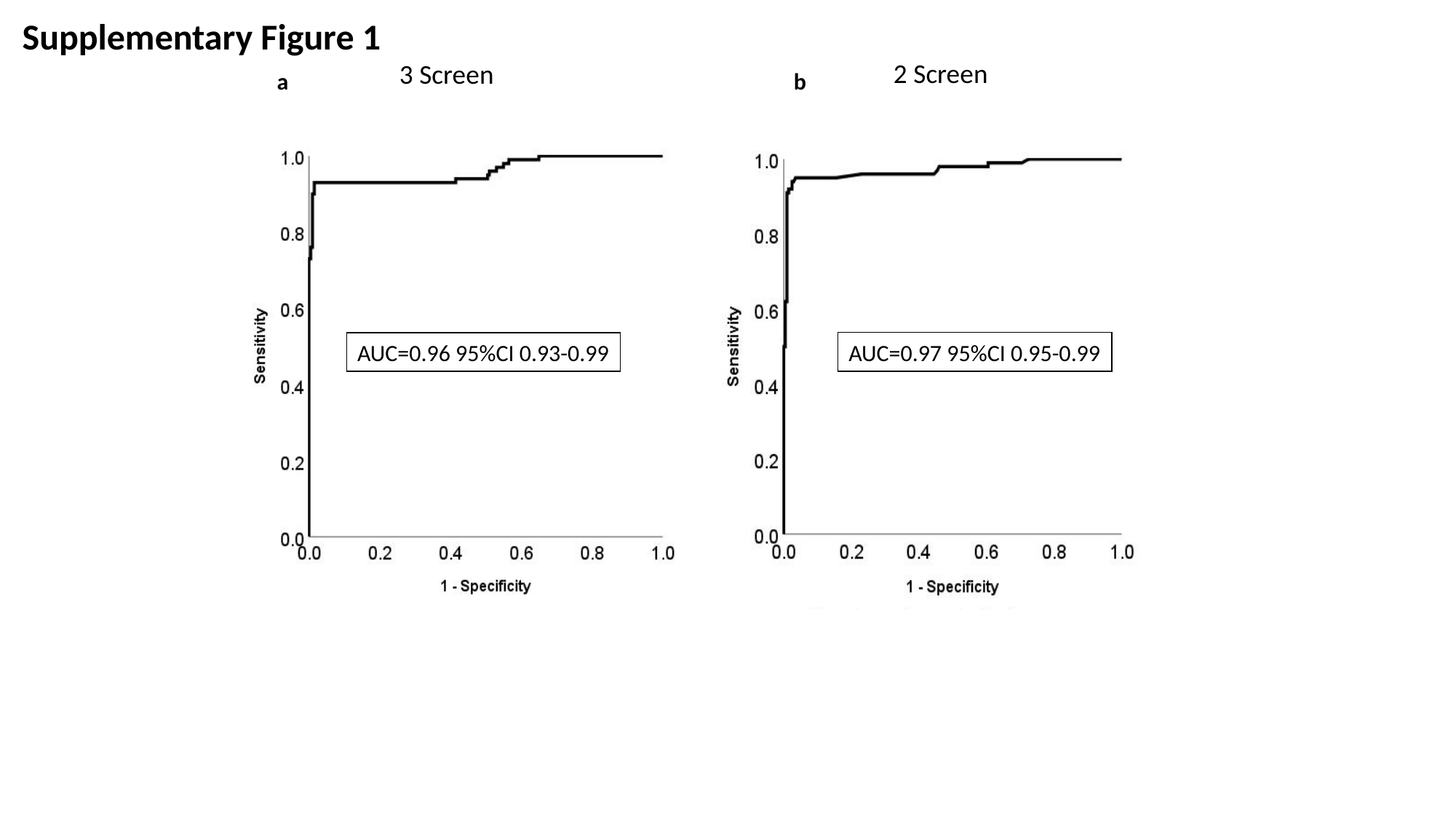

Supplementary Figure 1
2 Screen
3 Screen
a
b
AUC=0.97 95%CI 0.95-0.99
AUC=0.96 95%CI 0.93-0.99

## Slide 3
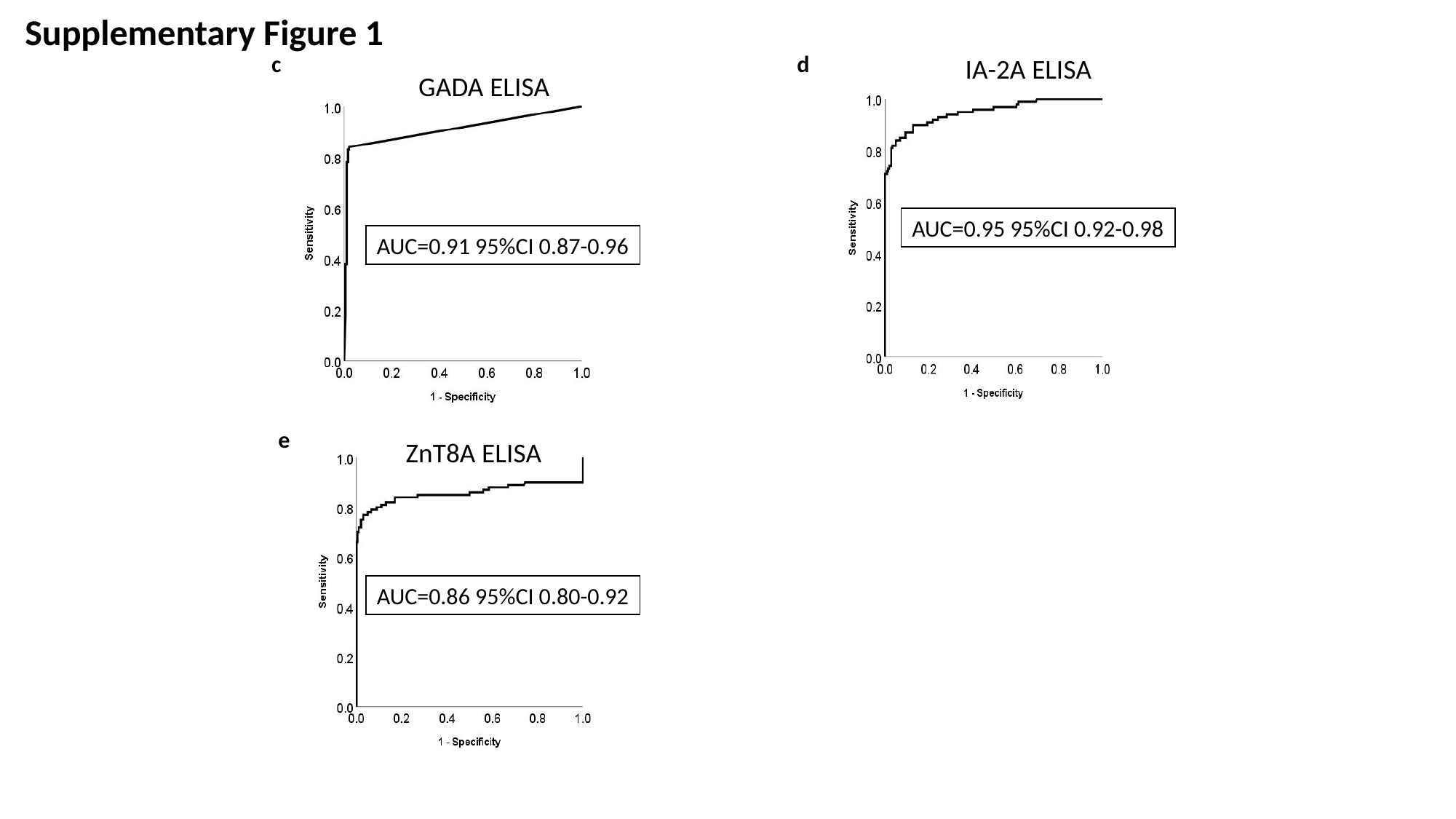

Supplementary Figure 1
c
d
IA-2A ELISA
GADA ELISA
AUC=0.95 95%CI 0.92-0.98
AUC=0.91 95%CI 0.87-0.96
e
ZnT8A ELISA
AUC=0.86 95%CI 0.80-0.92

## Slide 4
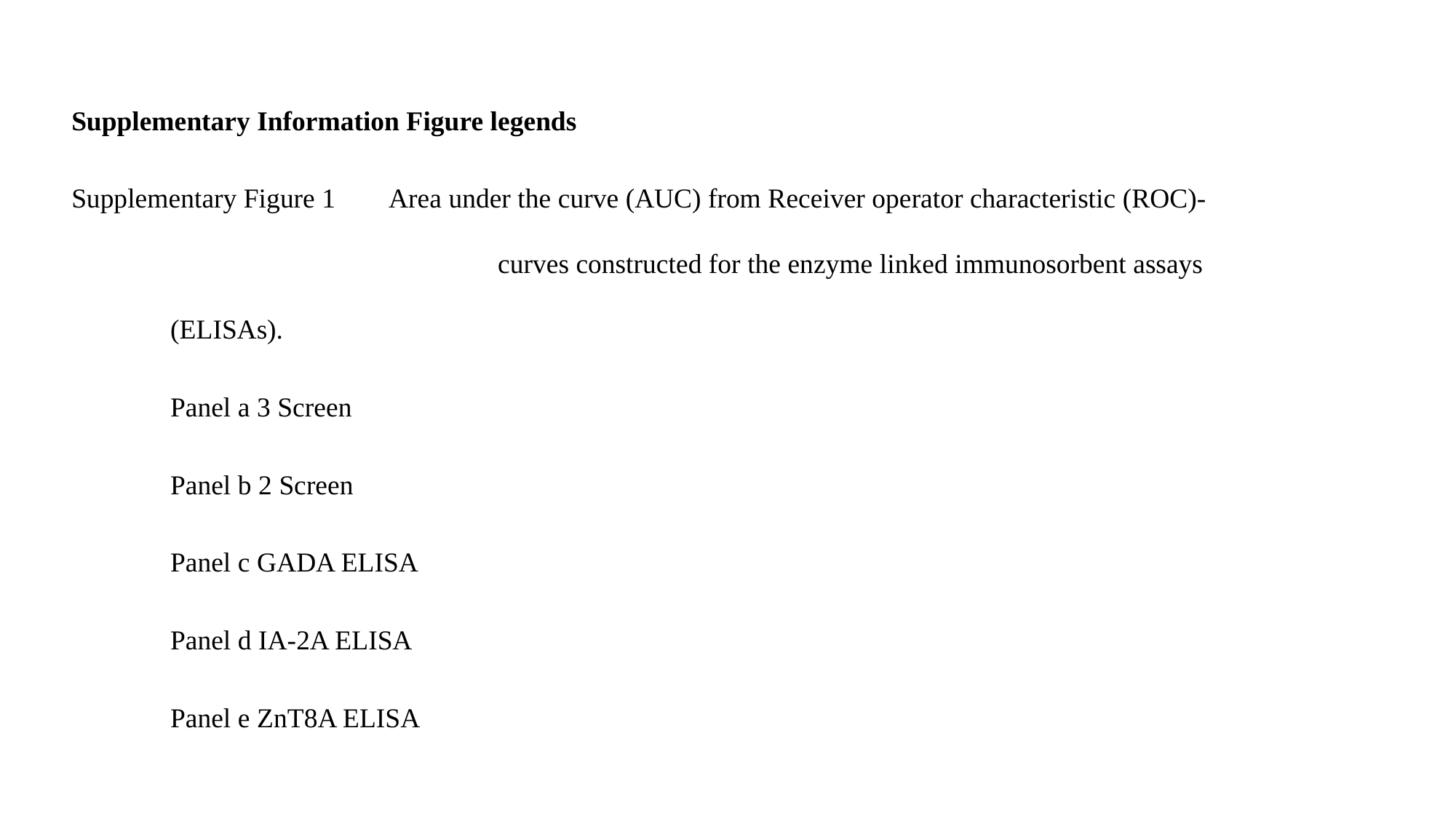

Supplementary Information Figure legends
Supplementary Figure 1 	Area under the curve (AUC) from Receiver operator characteristic (ROC)-				curves constructed for the enzyme linked immunosorbent assays (ELISAs).
Panel a 3 Screen
Panel b 2 Screen
Panel c GADA ELISA
Panel d IA-2A ELISA
Panel e ZnT8A ELISA

## Slide 5
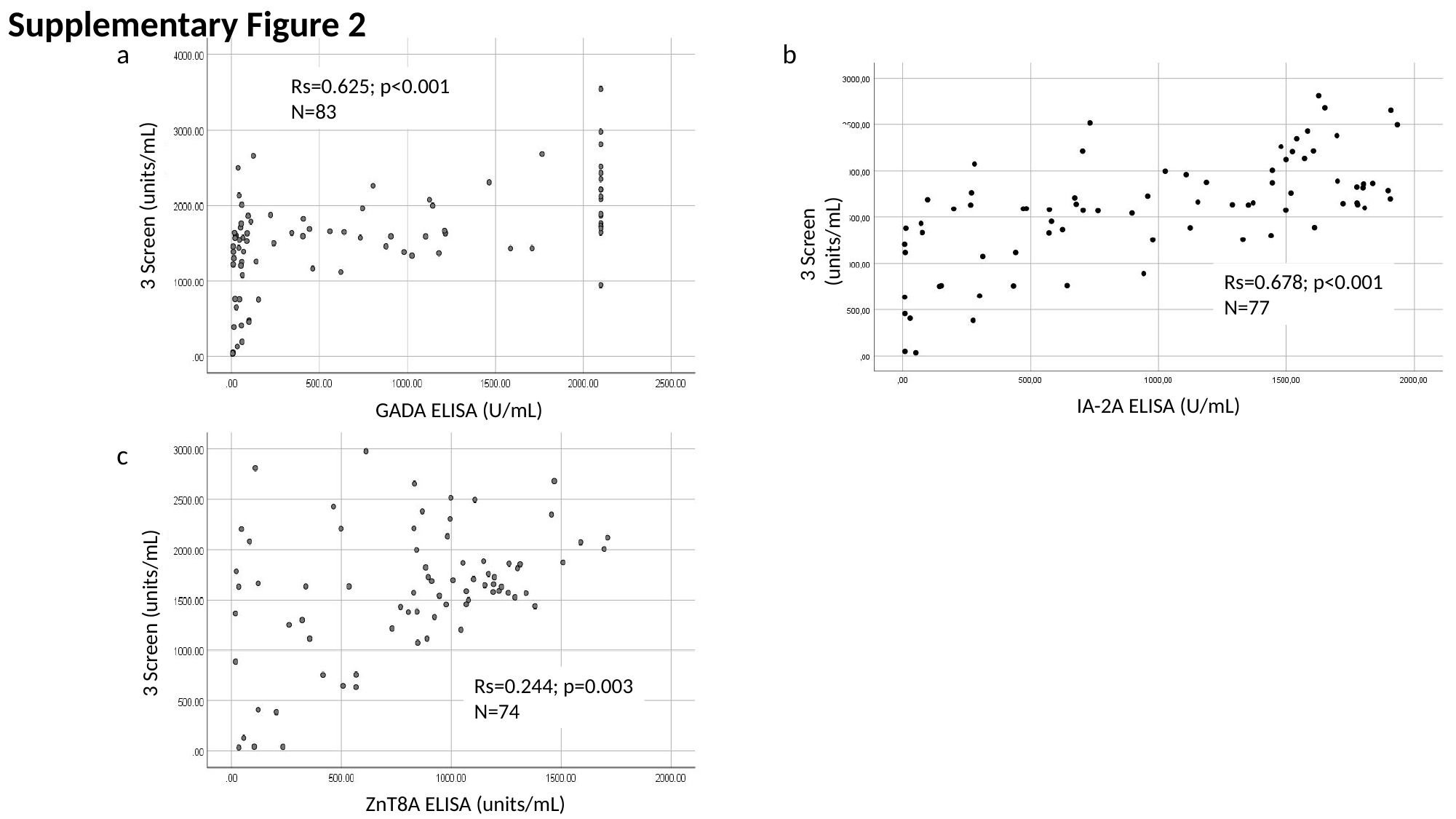

Supplementary Figure 2
a
b
Rs=0.625; p<0.001
N=83
 3 Screen (units/mL)
 3 Screen (units/mL)
Rs=0.678; p<0.001
N=77
IA-2A ELISA (U/mL)
GADA ELISA (U/mL)
c
3 Screen (units/mL)
Rs=0.244; p=0.003
N=74
ZnT8A ELISA (units/mL)

## Slide 6
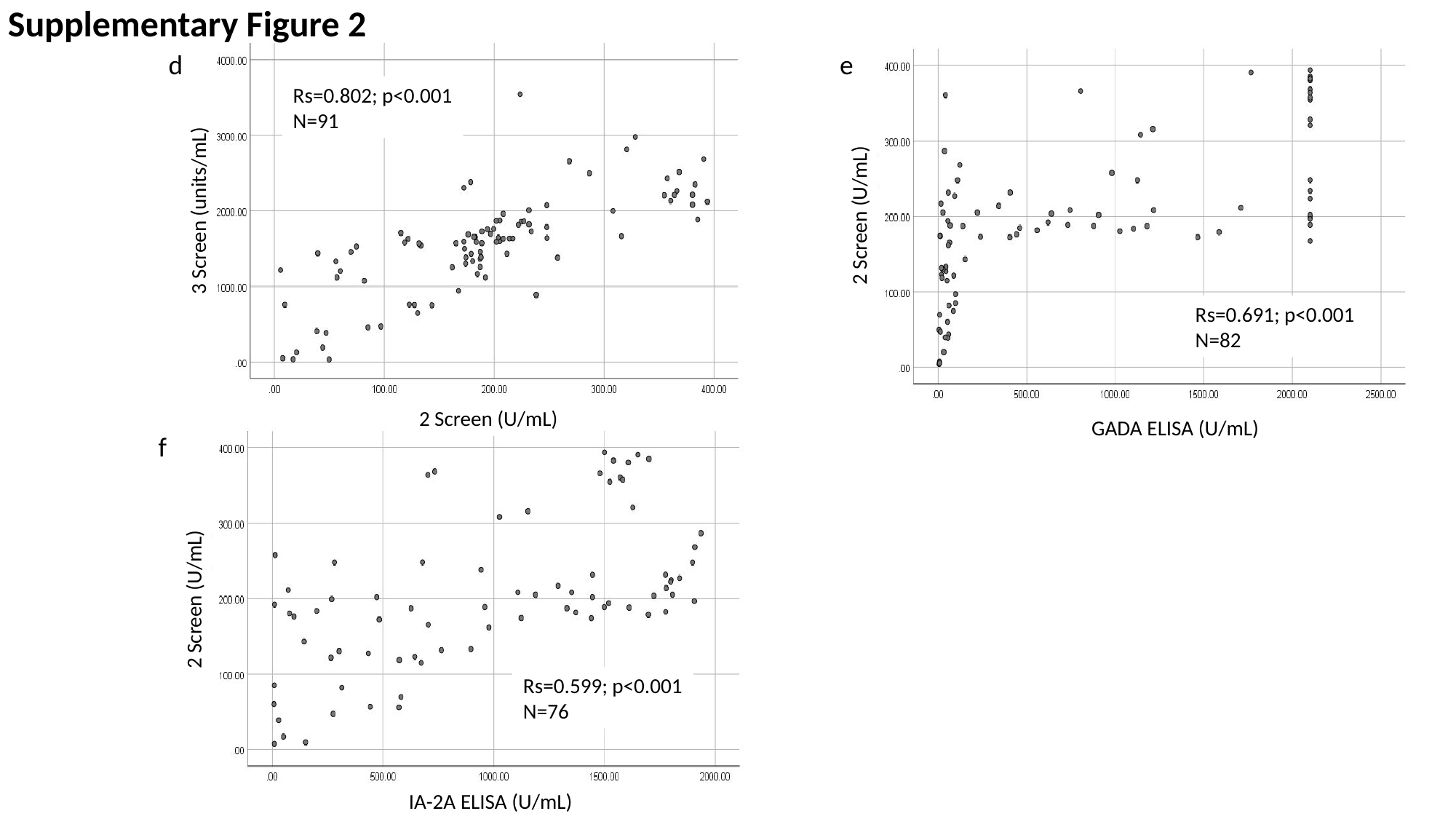

Supplementary Figure 2
d
e
Rs=0.802; p<0.001
N=91
3 Screen (units/mL)
2 Screen (U/mL)
Rs=0.691; p<0.001
N=82
2 Screen (U/mL)
GADA ELISA (U/mL)
f
2 Screen (U/mL)
Rs=0.599; p<0.001
N=76
IA-2A ELISA (U/mL)

## Slide 7
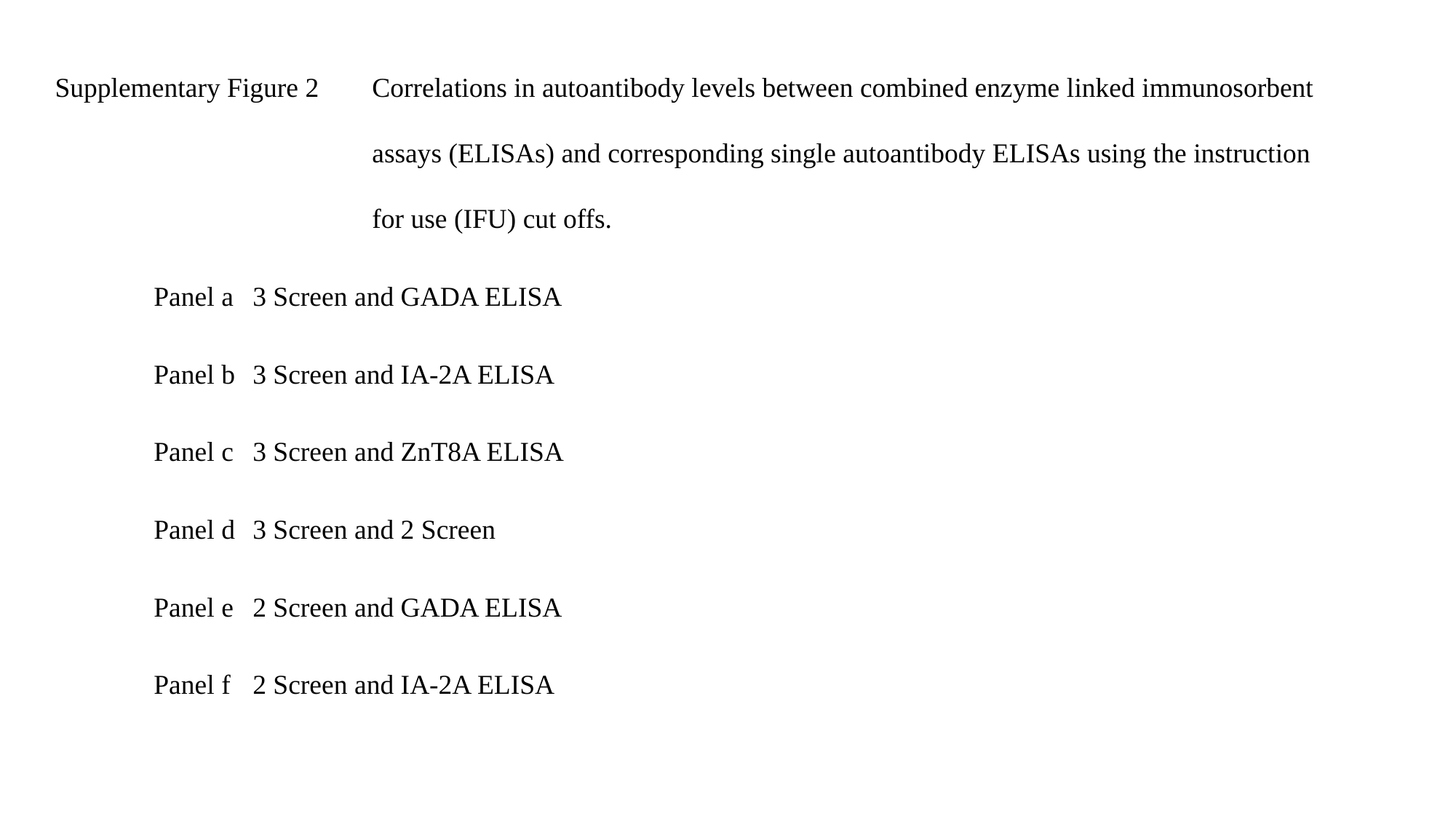

Supplementary Figure 2	Correlations in autoantibody levels between combined enzyme linked immunosorbent 			assays (ELISAs) and corresponding single autoantibody ELISAs using the instruction 			for use (IFU) cut offs.
Panel a	3 Screen and GADA ELISA
Panel b	3 Screen and IA-2A ELISA
Panel c	3 Screen and ZnT8A ELISA
Panel d	3 Screen and 2 Screen
Panel e	2 Screen and GADA ELISA
Panel f	2 Screen and IA-2A ELISA

## Slide 8
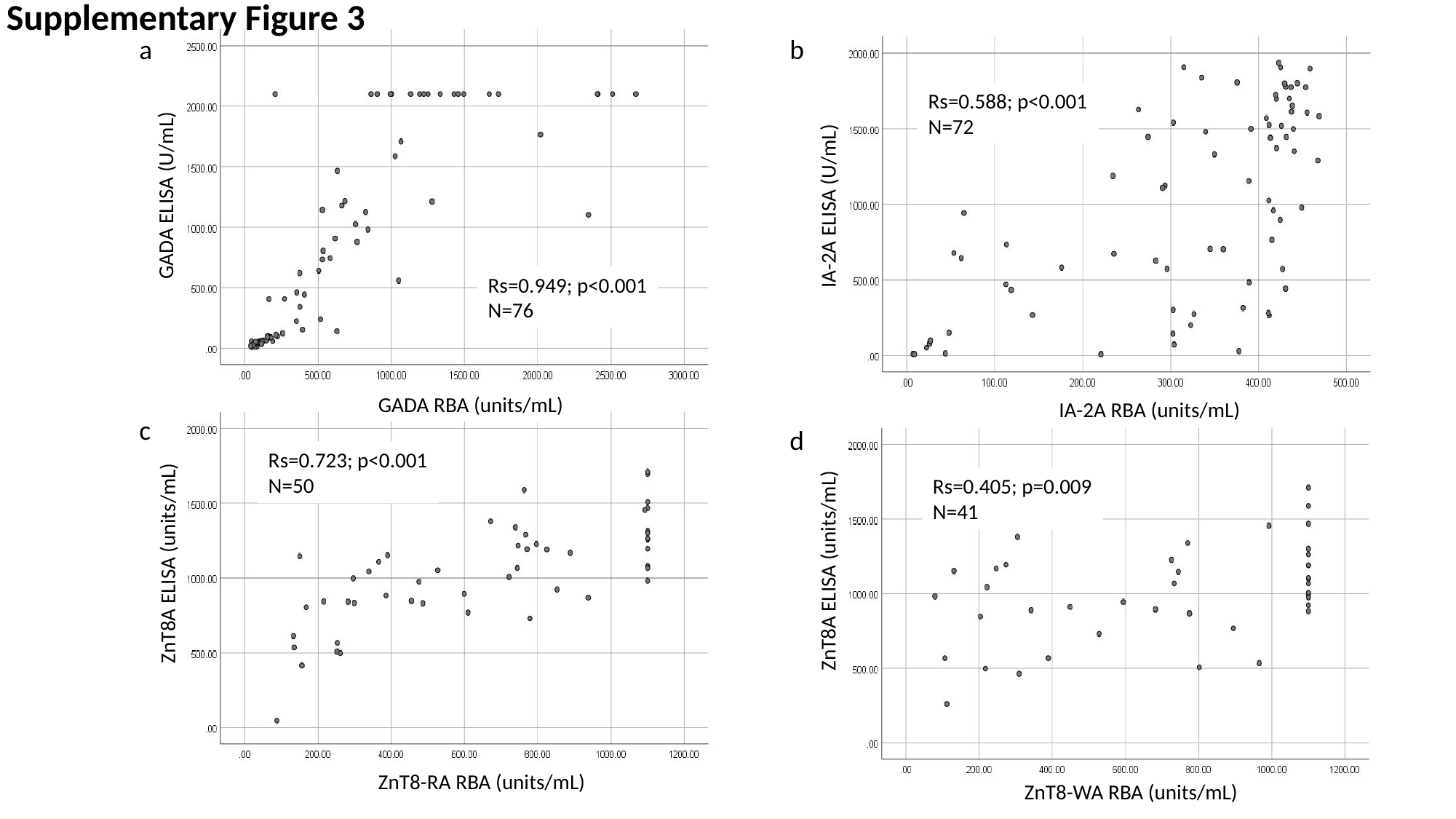

Supplementary Figure 3
a
b
Rs=0.588; p<0.001
N=72
GADA ELISA (U/mL)
IA-2A ELISA (U/mL)
IA-2A ELISA (U/mL)
Rs=0.949; p<0.001
N=76
GADA RBA (units/mL)
IA-2A RBA (units/mL)
c
d
Rs=0.723; p<0.001
N=50
Rs=0.405; p=0.009
N=41
ZnT8A ELISA (units/mL)
ZnT8A ELISA (units/mL)
ZnT8-RA RBA (units/mL)
ZnT8-WA RBA (units/mL)

## Slide 9
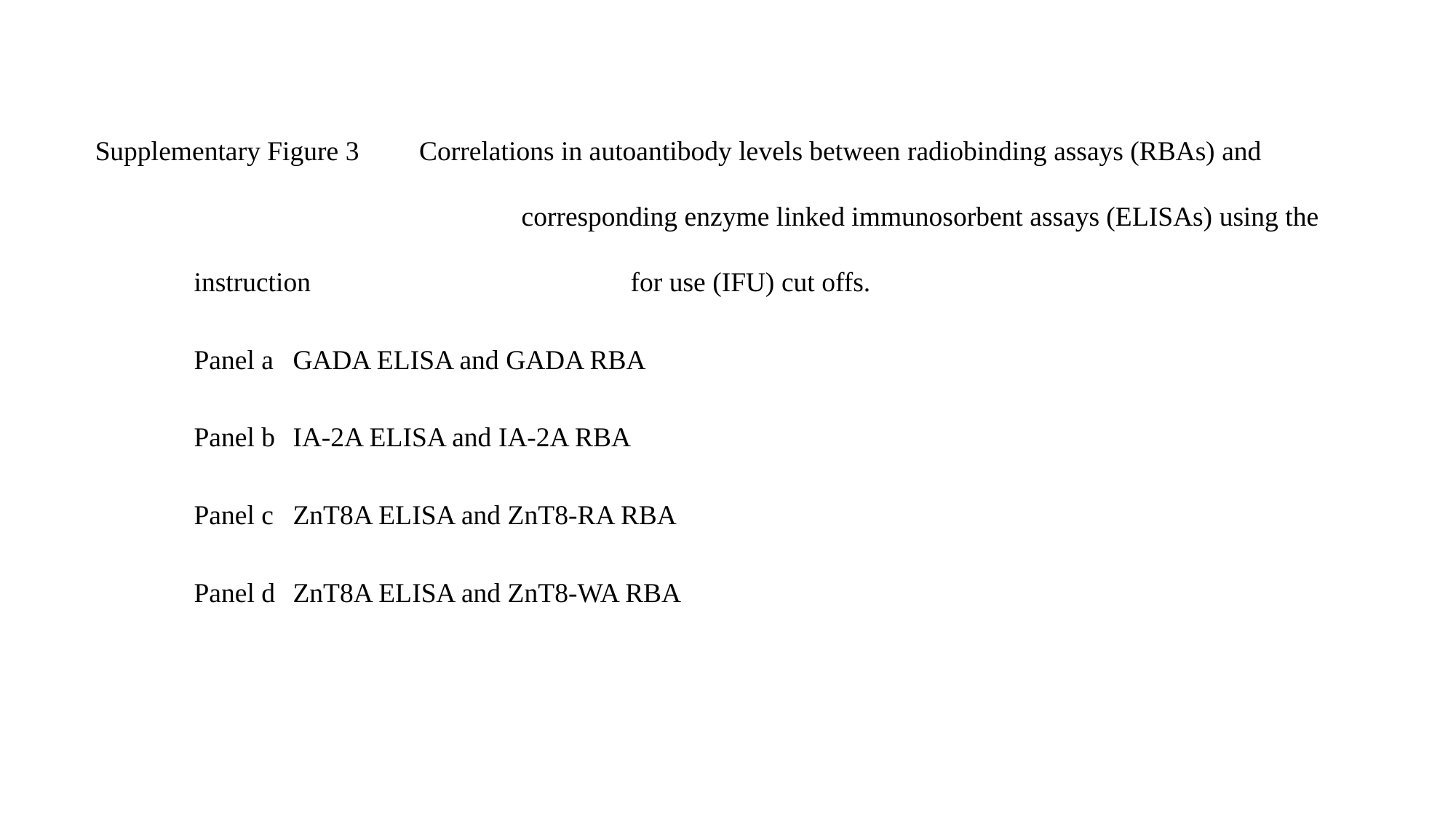

Supplementary Figure 3	 Correlations in autoantibody levels between radiobinding assays (RBAs) and 				corresponding enzyme linked immunosorbent assays (ELISAs) using the instruction 			for use (IFU) cut offs.
Panel a	GADA ELISA and GADA RBA
Panel b 	IA-2A ELISA and IA-2A RBA
Panel c	ZnT8A ELISA and ZnT8-RA RBA
Panel d 	ZnT8A ELISA and ZnT8-WA RBA
